# Development of cancer prognostic signature based on pan-cancer proteomics

**DOI:** 10.1080/21655979.2020.1847398

**Published:** 2020-12-08

**Authors:** Weiguo Huang, Jianhui Chen, Wanqing Weng, Yukai Xiang, Hongqi Shi, Yunfeng Shan

**Affiliations:** a Department of Hepatobiliary Surgery, The First Affiliated Hospital, Wenzhou Medical University, Wenzhou, China

**Keywords:** Proteomics, pan-cancer, biomarker, prognosis, differentially expressed proteins

## Abstract

Utilizing genomic data to predict cancer prognosis was insufficient. Proteomics can improve our understanding of the etiology and progression of cancer and improve the assessment of cancer prognosis. And the Clinical Proteomic Tumor Analysis Consortium (CPTAC) has generated extensive proteomics data of the vast majority of tumors. Based on CPTAC, we can perform a proteomic pan-carcinoma analysis. We collected the proteomics data and clinical features of cancer patients from CPTAC. Then, we screened 69 differentially expressed proteins (DEPs) with R software in five cancers: hepatocellular carcinoma (HCC), children’s brain tumor tissue consortium (CBTTC), clear cell renal cell carcinoma (CCRC), lung adenocarcinoma (LUAD) and uterine corpus endometrial carcinoma (UCEC). GO and KEGG analysis were performed to clarify the function of these proteins. We also identified their interactions. The DEPs-based prognostic model for predicting over survival was identified by least absolute shrinkage and selection operator (LASSO)-Cox regression model in training cohort. Then, we used the time-dependent receiver operating characteristics analysis to evaluate the ability of the prognostic model to predict overall survival and validated it in validation cohort. The results showed that the DEPs-based prognostic model could accurately and effectively predict the survival rate of most cancers.

## Introduction

As the most prevalent fatal disease, cancer ranked second in all mortality worldwide in 2017 [1]. And the death rate of cancer was increasing year by year, cancer deaths increased from 7.62 million in 2007 to 9.56 million in 2017. In 2018, 18.1 million people worldwide have been diagnosed with various types of cancer [2]. Despite the significant progress in treatment, timely diagnosis and high cost of treatment make it impossible to obtain effective treatment, which was still the reason for the low 5-year survival rate of most cancers [3]. In order to develop optimal anti-cancer treatment protocols and elucidate the mechanism of tumorigenesis, it is essential to estimate the prognosis of tumor patients [4]. Although many studies used RNA sequence data from the Cancer Genome Atlas (TCGA) and Genotype-Tissue Expression (GTEx) to evidence many tumor prognostic biomarkers and construct many prognostic models [5,6], utilizing genomic data to predict cancer prognosis was insufficient and imprecise, because molecular drivers of cancer were derived not just from DNA alterations alone, but from protein expression, modification, and activity at the metabolic level [7].

It is widely acknowledged that tumor cells were characterized by rapid generation and 16abnormal proliferation. Hence, tumor tissues would regulate the expression of proteins and promote the production of proteins associated with cancer progression [8]. Moreover, proteins were the functional effectors of cellular processes as well as the targets for a vast majority of therapeutics [9]. Therefore, the study of proteomics can improve our understanding of cancer etiology and progression as well as heighten the assessment of cancer prognosis [10]. Although most previous studies have focused on the effects of individual-specific protein on cancer prognosis [11–13], cancer is a heterogeneity disease that does not only involve individual protein but also interactions among proteins of different function. The Clinical Proteomic Tumor Analysis Consortium (CPTAC) project had generated a great deal of proteomics data of the vast majority of tumors by mass spectrometry [14]. Based on the proteomics data from CPTAC, we expect to combine multiple proteins to construct a pan-cancer prognostic model.

In current study, we screened out differentially expressed proteins (DEPs) in five cancers: hepatocellular carcinoma (HCC), uterine corpus endometrial carcinoma (UCEC), children’s brain tumor tissue consortium (CBTTC), lung adenocarcinoma (LUAD) and clear cell renal cell carcinoma (CCRC). Next, we explored the role of the differentially expressed proteins in cancer and the relationships among them. Furthermore, the DEPs-based survival-predictor model was also developed for predicting survival rates for the vast majority of cancers.

## Methods

### Patient datasets

The proteomic data of HCC, CBTTC, CCRC, LUAD and UCEC were extracted from the CPTAC [14] (https://proteomics.cancer.gov/programs/cptac) in November 2019.

### Identification of DEPs between tumor tissues and adjacent nontumorous tissues

For the proteomic data from CPTAC, background correction, quantile normalization, and batch normalization were performed using R software (version 3.6.1). The protein expression values of these five cancers were normalized by the ‘sva’ package. The bioconductor (http://www.bioconductor.org) package ‘limma’ was employed for DEP screening. A |log2Fold Change|>1 and an adjusted *P* value <0.05 were set as cutoff criteria.

### Functional enrichment analyses

We performed KEGG (Kyoto Encyclopedia of Genes and Genomes) analysis and Gene ontology (GO) analysis using R package ‘enrichplot,’ ‘enrichplot,’ ‘GOplot.’

### PPI network construction

The PPI network of DEPs was performed by STRING [15] (https://string-db.org/) and a combined score >0.9 (high confidence) was set as the cutoff criterion. Using cytoscape online software (http://www.cytoscape.org/) to visualize the results from STRING.

### Construction of DEPs-based classifiers

Based on univariate Cox regression models, we identify single DEP as independent prognostic DEPs for OS with p-value<0.05. The least absolute shrinkage and selection operator (LASSO)-Cox regression model [16] was used to identify the most accurate predictive DEPs for OS. The correlation of each prognostic DEPs was performed by R package ‘ggcorrplot,’ ‘statn.’

### Predictive performance of the DEPs-based classifiers

The patient’s risk score is obtained by multiplying the expression of DEPs in LASSO by their respective coefficients. And the patients were stratified into two risk-groups by median. The survival was analyzed by the Kaplan–Meier log-rank analysis. The time-dependent receiver operating characteristics (tdROC) analysis was used to assess performance of single DEP and classifiers through the ‘timeROC’ package of R software. The area under the curve (AUC) of tdROC reflected predictive accuracy. P-values <0.05 were considered statistically significant.

### Data analysis

The Student’s t-test, Wilcoxon test, and other data processing were completed by SPSS 19.0. Kaplan-Meier analysis is calculated by the ‘survminer’ package of R software. When all the hypotheses are P < 0.05, the difference is statistically significant.

## Results

### Differentially expressed proteins in five cancers

**Figure 1.**
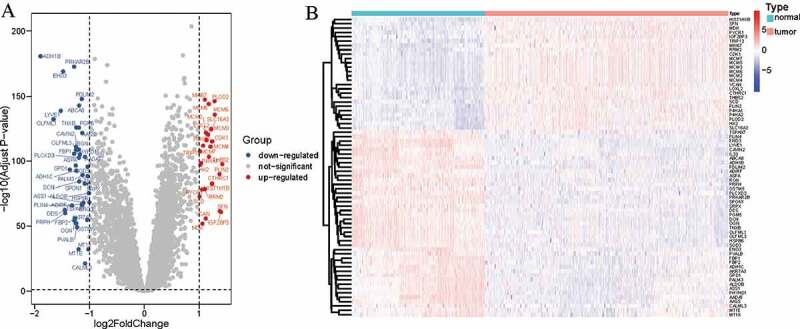
Identification of DEPs in five cancers. DEPs were defined with P-value < 0.05 and |log2(Fold Change)|>1. (a) Volcano plots of proteins with normalized expression alteration in all five cancers; (b) Heatmap of the DEPs (n = 69) in all five cancers. DEPs, differentially expressed proteins

Firstly, we acquired five types of cancer of proteomic data sets from the CPTAC data portal, which contained HCC, CBTTC, CCRC, LUAD and UCEC. According to the criteria of log2 | FoldChange |> 1 and FDR <0.05, we identified 69 differentially expressed proteins (DEPs) between tumor tissues and adjacent nontumorous tissues using ‘limma,’ and then plotted volcano and heat maps (Figure 1a,b). Among the 69 proteins, 26 proteins expression were upregulated in cancerous tissues such as Cyclin-dependent kina (CDK1) and Proliferation marker protein Ki-67 (MKI67), while 43 proteins were down-regulated in cancerous tissues such as Beta-enolase (ENO3) and Glycerol-3-phosphate dehydrogenase [NAD(+)] (GPD1) (Figure 1b).

### GO analysis and KEGG analysis

**Figure 2.**
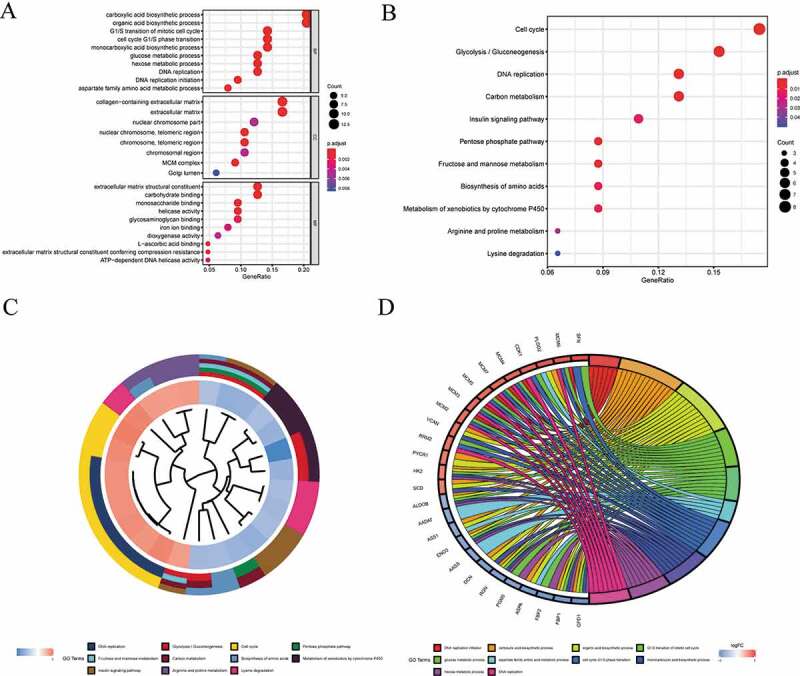
GO analysis and KEGG analysis of DEPs. (a) The functions of the 69 DEPs identified cover three main categories: BP, CC, MF; (b) based on KEGG pathway, 11 enriched pathways with lowest P-value were displayed; (c) (d) GO cluster diagram and GO chord diagram of the 69 DEPs. DEPs, differentially expressed proteins; BP, biological processes; CC, cellular contents; MF, molecular functions; GO, gene ontology

In order to explore the role of the 69 DEPs in tumors, we conducted GO analysis and KEGG analysis. And the 69 DEPs were mainly associated with the following biological processes: carboxylic acid biosynthetic process, organic acid biosynthetic process, G1/S transition of mitotic cell cycle, cell cycle G1/S phase transition, monocarboxylic acid biosynthetic process, glucose metabolic process, hexose metabolic process, and DNA replication (Figure 2a). The results also indicated that the DEPs were mainly associated with the following cellular contents: nuclear chromosome part, extracellular matrix, telomeric region and MCM complex (Figure 2a). Besides, the DEPs were related to molecular functions, such as extracellular matrix structural constituent, carbohydrate binding, helicase activity and monosaccharide binding (Figure 2a). Similar to GO analysis, KEGG analysis showed the DEPs primarily contributed to the following pathways: Cell cycle, Glycolysis/Gluconeogenesis, DNA replication, Carbon metabolism, Pentose phosphate pathway and Fructose and mannose metabolism (Figure 2b). Furthermore, combining GO cluster diagram and GO chord diagram, we found that the parts of DEPs involved in DNA replication, Cell cycle and Arginine and proline metabolism were mainly high-expressed, and others associated with these GO terms such as Carbon metabolism and Fructose and mannose metabolism were both highly and poorly expressed (Figure 2c,d).

### DEPs interaction clusters common across five cancers

**Figure 3.**
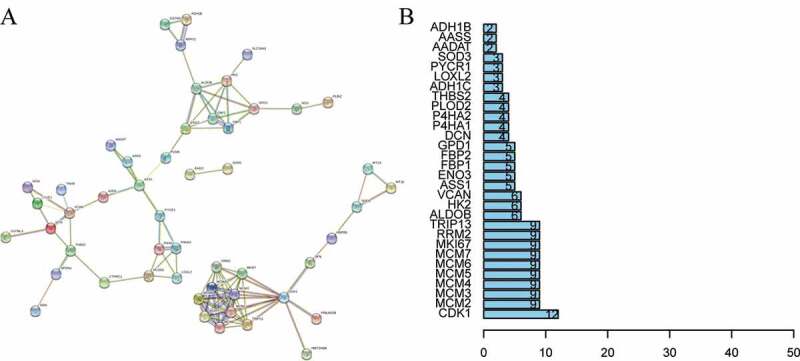
PPI network. (a) Interactions among 69 DEPs were detected after removing isolated proteins; (b) the number of interactions between each protein and other proteins. PPI, protein-protein interaction; DEPs, differentially expressed proteins

The 69 DEPs were used for the network analysis and almost half the DEPs formed an interaction network after eliminating proteins that acted independently (Figure 3a). And these interacting proteins were roughly separated into four groups with CDK1, ENO3, Argininosuccinate synthase (ASS1) and Versican core protein (VCAN) as the cores (Figure 3a,b). CDK1 was observed to be the key hub protein that interacted with DNA replication licensing factor MCM2 (MCM2), DNA replication licensing factor MCM3 (MCM3), DNA replication licensing factor MCM4 (MCM4), DNA replication licensing factor MCM5 (MCM5), DNA replication licensing factor MCM6 (MCM6), DNA replication licensing factor MCM7 (MCM7), MKI67, Ribonucleoside-diphosphate reductase subunit M2 (RRM2), TRIP13, 14-3-3 protein sigma (SFN), Histone H1.5 (HIST1H1B), cAMP-dependent protein kinase type II-beta (PRKAR2B). ENO3 interacted with Hexokinase-2 (HK2), Fructose-1,6-bisphosphatase isozyme 1 (FBP1), Fructose-1,6-bisphosphatase isozyme 2 (FBP2), Fructose-bisphosphate aldolase B (ALDOB), and Phosphoglucomutase-like protein 5 (PGM5). VCAN interacted with Aspartoacylase (ASPA), Decorin (DCN), Thrombospondin-2 (THBS2), Tenascin-X (TNXB), Lymphatic vessel endothelial hyaluronic acid receptor 1 (LYVE1) and Mimecan (OGN). ASS1 interacted with PGM5, ASPA, Alpha-aminoadipate aminotransferase (AADAT), pyrroline-5-carboxylate reductase 1 (PYCR1), and Alpha-aminoadipic semialdehyde synthase (AASS).

### The effect of individual DEPs on survival

To explore the effect of these proteins on cancer prognosis, Kaplan-Meier survival analyses were performed using individual protein. Based on the median value of each DEP expression, we divided the cancer patients into two clusters: high protein level and low protein level. Then, we defined four types of cancer as the training cohort: HCC, CCRC, LUAD, and UCEC; and defined CBTTC as validation cohort. We counted the OS of patients from the training cohort. As shown in Figure S1, only 10 proteins out of 69 DEPs were statistically significant in the survival analysis (P < 0.05). Patients whose cancerous tissue expressed higher levels of one of RRM2, Procollagen-lysine,2-oxoglutarate 5-dioxygenase 2 (PLOD2), MKI67, MCM5, and CKD1 had lower survival rates (Figure S1A-E). And Patients whose cancerous tissue expressed higher levels of one of FBP1, FBP2, ENO3, GPD1, and ASS1 had higher survival rates (Figure S1F-J). Yet regrettably, receiver operating characteristic (ROC) analysis of them were unsatisfactory: RRM2 (1 year AUC = 0.622), PLOD2 (1 year AUC = 0.635), MKI67 (1 year AUC = 0.617), MCM5 (1 year AUC = 0.595), CKD1 (1 year AUC = 0.610), FBP1 (1 year AUC = 0.323), FBP2 (1 year AUC = 0.320), ENO3 (1 year AUC = 0.383), GPD1 (1 year AUC = 0.362), ASS1 (1 year AUC = 0.437) (Figure S2). Although 3 years AUC of PLOD2 reached 0.722, 1 year and 2 years AUC were unsatisfactory. In summary, although the 10 proteins can be used as biomarkers of cancer prognosis, none of them could accurately predict OS.

### DEPs-based survival-predictor model constructing

**Figure 4.**
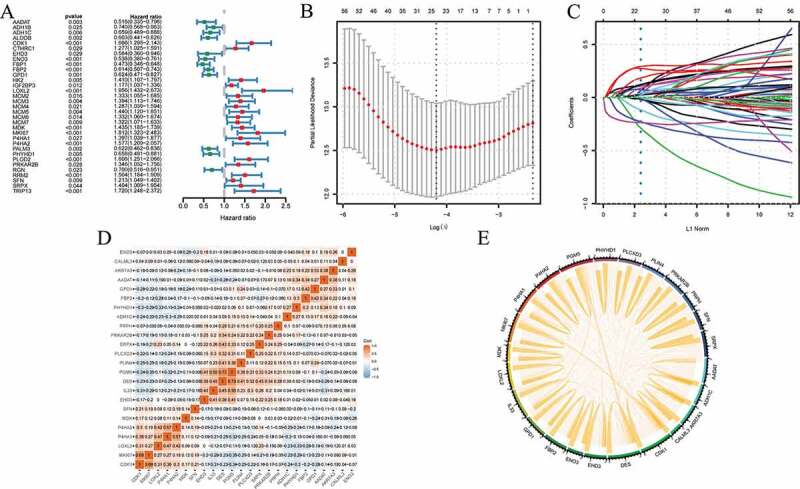
The survival-predictor model based on twenty-four-DEPs. (a) Univariate Cox analyses showed that 33 DEPs contributed to the OS in the training cohort; (b)(c) the LASSO regression model identified the 24 most accurate predictive DEPs in the training cohort; (d) (e) the expression relationship of the 24 DEPs was displayed. DEPs, differentially expressed proteins; OS, overall survival

For acquiring a more excellent model, multiple DEPs were combined to predict survival rates for cancer patients. We first conducted univariate Cox analyses in training cohort and found that 33 DEPs related to survival were identified (Figure 4a). Then, we used 69 DEPs to perform the LASSO Cox regression model in training cohort. Based on the results of the LASSO Cox regression model, 24 prognostic DEPs with non-zero regression coefficients were finally chosen as the potential prognostic biomarkers for the OS of cancer patients (Figure 4b,c). The detailed information of DEPs for constructing the prognostic signature was summarized in Table 1. The formula of the twenty-four-DEPs survival-predictor model was as follows: twenty-four-DEPs predictor model score = (0.303235530256179*MKI67)+(0.259559228152558*LOXL2)+(0.216349150569518*PLIN4)+(0.163857099694478*IL33)+(0.153385186100743*MDK)+(0.144674735753098*P4HA2)+(0.13190528953757*AKR7A3)+(0.121054348420759*PLCXD3)+(0.120550067398402*CDK1)+(0.077423785033028*SRPX)+(0.0692670634423047*PRPH)+(0.0633036451678804*PRKAR2B)+(0.0468066907473914*P4HA1)+(0.0467283261834873*CALML3)+(0.0342288464237997*SFN)+(0.00251963795312595*DES)-(0.021873780076824*PHYHD1)-(0.041717955104614*GPD1)-(0.0516581125556701*AADAT)-(0.0740355402044938*PGM5)-(0.165116242778278*ADH1C)-(0.245540744438086*FBP2)-(0.265391369780318*ENO3)-(0.388025693935519*EHD3). The correlationship of each protein in the 24-DEPs model was shown in Figure 4d,e. Among these proteins, the values of correlation between CDK1 and MKI67, P4HA2 and P4HA1, PGM5 and IL33, PGM5 and DES were all more than 0.5.

**Table 1.** The detailed information of differentially expressed proteins for constructing the prognostic signature

Protein name	Gene name	β
alpha-aminoadipate aminotransferase	AADAT	−0.051658113
Alcohol dehydrogenase 1 C	ADH1C	−0.165116243
Aflatoxin B1 aldehyde reductase member 3	AKR7A3	0.13190529
Calmodulin-like protein 3	CALML3	0.046728326
Cyclin-dependent kinase 1	CDK1	0.120550067
Desmin	DES	0.002519638
EH domain-containing protein 3	EHD3	−0.388025694
Beta-enolase	ENO3	−0.26539137
Fructose-1,6-bisphosphatase isozyme 2	FBP2	−0.245540744
Glycerol-3-phosphate dehydrogenase [NAD(+)]	GPD1	−0.041717955
Interleukin-33	IL33	0.1638571
Lysyl oxidase homolog 2	LOXL2	0.259559228
Midkine	MDK	0.153385186
Proliferation marker protein Ki-67	MKI67	0.30323553
Prolyl 4-hydroxylase subunit alpha-1	P4HA1	0.046806691
Prolyl 4-hydroxylase subunit alpha-2	P4HA2	0.144674736
Phosphoglucomutase-like protein 5	PGM5	−0.07403554
Phytanoyl-CoA dioxygenase domain-containing protein 1	PHYHD1	−0.02187378
PI-PLC X domain-containing protein 3	PLCXD3	0.121054348
Perilipin-4	PLIN4	0.216349151
cAMP-dependent protein kinase type II-beta regulatory subunit	PRKAR2B	0.063303645
Peripherin	PRPH	0.069267063
14-3-3 protein sigma	SFN	0.034228846
Sushi repeat-containing protein SRPX	SRPX	0.077423785

### Evaluation of the survival-predictor model

**Figure 5.**
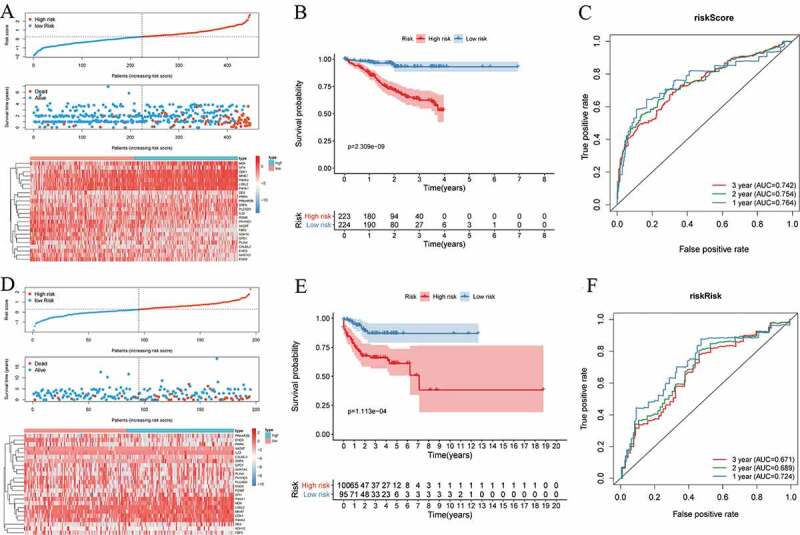
Time-dependent ROC curves and the survival analysis for the DEPs-based classifiers for OS in the training cohort and the validation cohort. (a,d) Cancer patients were divided into two groups by the median of risk score in the training cohort: High risk and Low risk; (b) Kaplan-Meier Survival analysis results indicated that the two groups had significantly different survival rates (p = 2.309e−09); (c) tdROC were applied to assess predictive accuracy for overall survival; (d) according to the same cutoff point cancer patients were also divided into two groups in the validation cohort; (e) Kaplan-Meier Survival analysis results indicated that the two groups had significantly different survival rates in the validation cohort (p = 1.113e−04); (f) tdROC were applied to assess predictive accuracy for overall survival. DEPs, differentially expressed proteins; OS, overall survival; tdROC, Time-dependent ROC

Based on the survival-predictor model, we evenly divided cancer patients into two groups by the median risk score cutoff point, which value is 0.250379: High risk and Low risk (Figure 5a). The patient information was shown in Tables 2 and 3. Furthermore, the expression heatmap of the 24 DEPs in high risk or low-risk group was shown in Figure 5a. We then estimated the accuracy of the 24-DEPs model on predicting survival. The Kaplan-Meier survival curves showed that survival rates were significantly lower in the High risk (P < 0.001) (Figure 5b). The ROC analysis showed the one, two, and three years AUC of the 24-DEPs survival-predictor model were 0.764, 0.754, and 0.742, respectively (Figure 5c). Remarkably, the AUC of the 24-DEPs survival-predictor model was more than the AUC of the 10 proteins described above (Figure S2). So, compared with a single protein as a predictor, the 24-DEPs survival prediction model had accurate and powerful prediction capability.

**Table 2.** Univariate and multivariate COX analyses of the DEPs-based classifier for OS

	Univariate analysis			Multivariate analysis		
Prognostic parameter	HR	95% CI	P value	HR	95% CI	P value
**CCRC**						
Age (> 65 vs. ≤ 65)	0.724	0.218–2.409	0.599			
Gender (male vs. female)	1.099	0.297–4.063	0.887			
Grade (G3&4 vs. G1&2)	1.901	0.603–5.993	0.273			
Tumor stage (III + IV vs. I + II)	2.031	1.224–3.369	**0.006**			
T classification (T3 + T4 vs. T1 + T2)	5.479	1.483–20.240	**0.011**			
N classification (N1 vs. N0) M classification (M1 vs. M0)	1.269 10.190	0.132–12.221 2.625–39.560	0.837 **0.001**	6.593	1.670–26.026	**0.007**
DEPs-based classifier (High vs. Low risk)	4.047	2.081–7.871	**<0.001**	4.438	1.704–11.561	**0.002**
**HCC**						
Age (> 65 vs. ≤ 65)	0.589	0.233–1.488	0.263			
Gender (male vs. female)	0.843	0.420–1.692	0.630			
Number of Tumors (Couple VS Single)	1.042	0.467–2.323	0.920			
Tumor thrombus (present vs. absent)	2.118	1.157–3.879	**0.015**	0.780	0.399–1.525	0.468
DEPs-based classifier (High vs. Low risk)	3.892	2.563–5.909	**<0.001**	3.114	1.911–5.073	**<0.001**
**LUAD**						
Age (> 65 vs. ≤ 65)	6.184	0.803–47.630	0.080			
Gender (male vs. female)	1.176	0.394–3.509	0.771			
Grade (G3&4 vs. G1&2)	0.259	0.026–2.540	0.246			
Tumor stage (III + IV vs. I + II)	7.518	2.428–23.283	**<0.001**	2.721	0.347–21.351	0.341
T classification (T3 + T4 vs. T1 + T2)	4.499	1.357–14.913	**0.014**	2.571	0.730–9.057	0.142
N classification (N1 vs. N0) M classification (M1 vs. M0)	5.400 0.048	1.658–17.585 0.00–10,988,250	**0.005** 0.757	1.973 6.593	0.257–15.160 1.670–26.026	0.514 **0.007**
Smoking history (Present vs. Absent)	2.310	0.754–7.070	0.143			
DEPs-based classifier (High vs. Low risk)	3.867	1.573–9.502	**0.003**	2.666	1.123–6.331	0.026
**UCEC**						
Age (> 65 vs. ≤ 65)	131.682	0.007–2,628,475	0.334			
Grade (G3&4 vs. G1&2)	3.042	0.190–48.629	0.432			
Tumor stage (III + IV vs. I + II)	5355.564	0–5.000E+18	0.626			
T classification (T3 + T4 vs. T1 + T2)	18.422	1.654–205.237	**0.018**	25,773.5	0–4.44094E+23	0.424
N classification (N1 vs. N0) M classification (M1 vs. M0)	0.039 35.500	0–1,243,745,686 2.220–567.557	0.793 0.012	0.000	0.000–1,102,092.5	0.424
DEPs-based classifier (High vs. Low risk)	13.430	1.983–183.437	**0.050**	216.159	0.003–14,902,987	0.344
**CBTTC**						
Age (> 65 vs. ≤ 65)	0.945	0.895–0.998	0.044	0.971	0.926–1.019	0.228
Gender (male vs. female)	0.708	0.387–1.293	0.261			
Surgery (Present vs. Absent)	0.102	0.047–0.222	**<0.001**	0.165	0.070–0.387	**<0.001**
DEPs-based classifier (High vs. Low risk)	13.430	1.983–183.437	**0.050**	2.173	1.229–3.843	**0.008**

HR, Hazard ratio; CI, confidence interval; DEPs, differentially expressed proteins; hepatocellular carcinoma, HCC; children’s brain tumor tissue consortium, CBTTC; clear cell renal cell carcinoma, CCRC; lung adenocarcinoma, LUAD; uterine corpus endometrial carcinoma, UCEC.

**Table 3.** Correlations between risk score of the DEPs-based classifier with overall survival and clinicopathological characteristics in five types of cancers

Clinicopathological variables	Number of patients	High Risk	Low Risk	P value
CCRC Age				
<65 (n, %)	67 (59.3%)	34 (30.1%)	33 (29.2%)	
≥65 (n, %)	46 (40.7%)	19 (16.8%)	27 (23.9%)	0.323
Gender				
Male (n, %)	30 (26.5%)	11 (9.7%)	19 (16.8%)	
Female (n, %)	83 (73.5%)	42 (37.2%)	41 (36.3%)	0.190
Histologic Grade				
G1+ G2 (n, %)	69 (61.1%)	27 (23.9%)	42 (37.2%)	
G3+ G4 (n, %) NA	44 (38.9%) 0	26 (23.0%)	18 (15.9%)	0.038
TNM staging system				
T1+ T2 (n, %)	72 (63.7%)	29 (25.7%)	43 (38.1%)	
T3+ T4 (n, %) NA	41 (36.3%) 0	24 (21.2%)	17 (15.0%)	0.061
N0 (n, %)	14 (77.8%)	9 (50.0%)	5 (27.8%)	
N1 (n, %) NA	4 (22.2%) 95	3(16.7%)	1(5.6%)	0.688
HCC				
Age				
<65 (n, %)	120 (85.1%)	85 (60.3%)	35 (24.8%)	
≥65 (n, %)	21 (14.9%)	16 (11.3%)	5 (3.5%)	0.615
Gender				
Male (n, %)	26 (18.4%)	19 (13.5%)	7 (5.0%)	
Female (n, %)	115 (81.6%)	82 (58.2%)	33 (23.4%)	0.856
Number of Tumors				
Single (n, %)	121(85.8%)	87 (61.7%)	34 (24.1%)	
Couple (n, %) NA	20 (14.2%) 0	14 (9.9%)	6 (4.3%)	0.861
LUAD				
Age				
<65 (n, %)	59 (57.8%)	29 (28.4%)	30 (29.4%)	
≥65 (n, %)	43 (42.2%)	18 (17.6%)	25 (24.5%)	0.466
Gender				
Male (n, %)	32 (31.4%)	12 (11.8%)	20 (19.6%)	
Female (n, %)	70 (68.6%)	35 (34.3%)	35 (34.3%)	0.240
Histologic Grade				
G1+ G2 (n, %)	62 (63.9%)	26 (26.8%)	36 (37.1%)	
G3+ G4 (n, %) NA	35 (36.1%) 5	18 (18.6%)	17 (17.5%)	0.367
TNM staging system				
T1+ T2 (n, %)	91 (89.2%)	39 (38.2%)	52 (51.0%)	
T3+ T4 (n, %) NA	11 (10.8%) 0	8 (7.8%)	3 (2.9%)	0.060
N0 (n, %)	70 (68.6%)	29 (28.4%)	41 (40.2%)	
N1 (n, %) NA	32 (31.4%) 0	18 (17.6%)	14 (13.7%)	0.163
M0 (n, %)	85 (97.7%)	42 (48.3%)	43 (49.4%)	
M1 (n, %) NA	2 (2.3%) 25	0 (.0%)	2 (2.3%)	0.167
Pathological stage				
I+ II (n, %)	81 (79.4%)	34 (33.3%)	47 (46.1%)	
III+IV (n, %) NA	21 (20.6%) 0	13 (12.7%)	8 (7.8%)	0.103
Smoking history				
Present (n, %)	56 (56.6%)	26 (26.3%)	30 (30.3%)	
Absent (n, %) N	43 (43.4%) 3	20 (20.2%)	23 (23.2%)	0.993
UCEC				
Age				
<65 (n, %)	56 (56.6%)	16 (16.2%)	40 (40.4%)	
≥65 (n, %)	43 (43.4%)	8 (8.1%)	35 (35.4%)	0.251
Histologic Grade				
G1+ G2 (n, %)	73 (75.3%)	13 (13.4%)	60 (61.9%)	
G3+ G4 (n, %) NA	24 (24.7%) 2	11 (11.3%)	13 (13.4%)	0.006
TNM staging system				
T1+ T2 (n, %)	88 (88.9%)	20 (20.2%)	68 (68.7%)	
T3+ T4 (n, %) NA	11 (11.1%) 0	4 (4.0%)	7 (7.1%)	0.320
N0 (n, %)	47 (85.5%)	9 (16.4%)	38 (69.1%)	
N1 (n, %) NA	8 (14.5%) 44	4 (7.3%)	4 (7.3%)	0.058
M0 (n, %)	71 (97.3%)	17 (23.3%)	54 (74.0%)	
M1 (n, %) NA	2 (2.7%) 26	1 (1.44%)	1 (1.4%)	0.399
FIGO stage				
I+ II (n, %)	82 (82.8%)	18 (18.2%)	64 (64.6%)	
III+IV (n, %) NA	17 (17.2%) 0	6 (6.1%)	11 (11.1%)	0.243
CBTTC				
Age				
<65 (n, %)	195 (99.5%)	100 (51.0%)	95 (48.5%)	
≥65 (n, %)	1 (0.5%)	1 (0.5%)	0 (0%)	0.331
Gender				
Male (n, %)	86 (43.9%)	44 (22.4%)	42 (21.4%)	
Female (n, %)	110 (56.1%)	57 (29.1%)	53 (27.0%)	0.927
Surgery				
Present (n, %)	163 (93.1%)	82 (46.7%)	81 (46.3%)	
Absent (n, %) N	12 (6.9%) 21	10 (5.7%)	2 (1.1%)	0.061

Hepatocellular carcinoma, HCC; children’s brain tumor tissue consortium, CBTTC; clear cell renal cell carcinoma, CCRC; lung adenocarcinoma, LUAD; uterine corpus endometrial carcinoma, UCEC.

In order to further validate the availability of this model, we used the same 24-DEPs survival-predictor model and cutoff point to cluster patients in validation cohort (CBTTC) (Figure 5d). And the survival analysis also indicated that high risk had a worse OS(P < 0.001) (Figure 5e). The result of the ROC analysis was also satisfactory: 1-year AUC = 0.724, 2-years AUC = 0.689, 3-years AUC = 0.671 (figure 5f). In conclusion, the 24 DEPs-based classifiers could accurately predict the survival not only in the training cohort, but also in the validation cohort.

## Discussion

As a complex disease, cancer involves not only in DNA alterations, but also in protein expression and modification [7].With technological improvements, CPTAC generates comprehensive mass spectrometry-based proteomic data for most cancers [14], which providing a unique opportunity for pan-cancerous proteomics research with sufficient data.

In current study, we firstly screened 69 differentially expressed proteins in five types of cancer tissue. More importantly, the expression trend of the DEPs was consistent in all five cancers, which indicated these proteins were not specific to any certain type of cancer. Among the DEPs, CDK1 played an important role in progression into mitotic phase, which could drive the cell cycle in all cell types [17]. Previous studies also showed that CDK1 expression was upregulated in a majority of tumor tissues, which correlated with the prognosis of cancer patients [18–20]. And MCM2, MCM3, MCM4, MCM5, MCM6, MCM7 formed the MiniChromosome Maintenance 2–7 complex, which was exported by the CDKs to trigger DNA replication [21]. In brief, CDK1 interacted with MCM2-7 complex to participate in the cell cycle, which was the same as the GO analysis and KEGG analysis. Furthermore, we found CDK1, as a key hub protein, interacted with other DEPs to form an interaction cluster. In addition to MCM2-7 complex, other proteins in the cluster also influenced the growth and division of tumor cells by participating in the cell cycle such as RRM2, PRKAR2B, and MKI67 [22–24]. Most DEPs related to the cell cycle were up-regulated, which was consistent with the vigorous growth and division of tumor cells. The 69 DEPs were involved not only in the cell cycle, but also in cell metabolism (Figure 2a,b). Since metabolic reprogramming was a well-established hallmark of cancer, alterations in metabolism-related proteins expression were common in tumors [25]. According to the Figure 3, metabolically related DEPs were roughly divided into two groups: carbohydrate metabolism-related proteins and amino acid metabolism-related proteins. ENO3, FBP1, FBP2, GPD1, and ALDOB were all glycolytic pathway-related proteins with inhibitory effects on tumor [26–29]. For instance, A LDOB disrupted redox homeostasis by reducing the levels of fructose 1,6-bisphosphate in tumor cells, which could inhibit tumor cell proliferation [27]. Previous research also showed that although gluconeogenesis was frequently suppressed in tumors, re-expression of gluconeogenesis enzymes such as FBP1 could inhibit tumor growth [29]. As an enzyme responsible for the biosynthesis of arginine in most body tissues, ASS1 was downregulated in multiple diverse cancers to reprogram arginine metabolism to make tumor cells more aggressive [30]. What’s more, according to our results, these metabolism-related proteins that inhibit cancer were also down-regulated. But also as a protein related to amino acid metabolism, PYCR1 was highly expressed to maintain the redox balance of tumor cells and prevent apoptosis by synthesizing proline [31]. Despite the DEPs associated with metabolism and cell proliferation, quite a few DEPs were associated with the extracellular matrix. As a large extracellular matrix proteoglycan, VCAN regulated proliferation, invasion, and metastasis adhesion in a vast majority of tumor cells, and VCAN expression was associated with poor prognosis in most cancers [32–34]. THBS2 was also an extracellular matrix protein and promoted cell migration and angiogenesis [35]. Distinguished with VCAN and THBS2, though DCN was associated with the extracellular matrix, it could antagonize many tyrosine kinase receptors to inhibit tumor development and progression [36]. According to these results, the four DEPs interaction clusters manifested that one cluster was involved in cell growth and division, one in carbohydrate metabolism, one in amino acid metabolism, and the rest in the extracellular matrix regulation. To summarize, the functions of the 69 DEPs fell into three main categories: cell proliferation and division, cellular metabolism, and extracellular matrix regulation.

In the following step, we performed Kaplan-Meier survival analyses of 69 DEPs one by one and found that only 10 DEPs were significantly correlated with survival for multiple cancer. Of the 10 proteins, the preceding text showed that some studies identified RRM2, PLOD2, MKI67, MCM5, and CKD1 promoted cancer progression and FBP1, FBP2, ENO3, GPD1, and ASS1 inhibited cancer progression, which was consistent with our results (Figure S1). Nevertheless, this traditional way of concentrating on molecular biomarkers such as single protein has not been successful; because the development and progression of cancers were primarily accomplished by a set of biomolecules, rather than the dysfunction of an individual molecule [37,38]. As shown in Figure S2, the accuracy of the 10 DEPs in predicting the prognosis of cancers was not high. Therefore, according to the LASSO regression method, we determined 24 DEPs: MKI67, LOXL2, PLIN4, IL33, MDK, P4HA2, AKR7A3, PLCXD3, CDK1, SRPX, PRPH, PRKAR2B, P4HA1, CALML3, SFN, DES, PHYHD1, GPD1, AADAT, PGM5, ADH1C, FBP2, ENO3, EHD3. In accordance with the above classification, among the 24 proteins, CDK1, SFN, PRKAR2B, MKI67 and MDK were involved in the cell cycle [17,24,39]; AKR7A3, GPD1, ENO3, FBP2, AADAT, PGM5 and ADH1C were involved in cell metabolism [26,28,40]; LOXL2, P4HA1, P4HA2, SPRX, DES, PRPH and CALML3 were involved in construction and regulation of extracellular matrix [41–43]. And most of these proteins have been identified to contribute to prognosis of many cancers [19,26,41–44]. Although IL33 and EHD3 did not belong to any of the three groups mentioned above, some researches showed that they could inhibit the proliferation of tumor cells [45,46]. In addition to these widely studied proteins, there were still several proteins whose roles in cancer were unclear such as PLCX3, PHYHD1 and PLIN3, which provided a new direction for cancer research. Although no research had yet explored the specific ways in which they interacted, according to correlation analysis, PGM5 was related to IL33 and DES. Therefore, we inferred that PGM5 may be involved in the regulation of tumor inflammation and extracellular matrix by regulating metabolism. Tumor immune microenvironment was closely related to tumor prognosis, and NK cells and T cells are the main anti-tumor cells, which were associated with cancer immune evasion [47–49]. Among the 24 DEPs, IL33 and EHD3 were associated NK cell and played an important role in TCR-mediated T cell functions [50,51]. Edwin Wang et al proposed a cancer hallmark network framework for modeling genome sequencing data associated clinical phenotypes [52,53]. And most of the 24 DEPs (CDK1, SFN, PRKAR2B, MKI67 and MDK involved in the cell cycle; LOXL2, P4HA1, P4HA2, SPRX, DES, PRPH and CALML3 involved in construction and regulation of extracellular matrix; IL33 and EHD3 may involve in immune) were linked to cancer hallmarks. Therefore, these DEPs could add to our understanding of tumor evolution and tumorigenesis and be helpful for predicting tumors’ evolutionary paths and clinical phenotypes. Based on the 24 DEPs-based classification, we divided the cancer patients into two groups in training cohort. The Kaplan-Meier survival analysis and the ROC analysis showed that the 24-DEPs survival-predictor model was better predictor than single protein (Figure 5b,c). We further verified the correctness of this grouping method in validation cohort and the two groups also showed significantly different survival rates (Figure 5e). Therefore, the DEPs-based survival-predictor model showed excellent survival prediction effect and is applicable to most cancers, which will contribute to therapeutic decision-making.

Yet, there are several limitations in this study. Firstly, this study mainly explored the effect of the differentially expressed proteins on predicting the OS of multiple cancers. It will inevitably be interesting to combine proteomics with genomics and even metabonomics to predict pan-cancer OS in the future. Secondly, the current study was a retrospective study utilizing the CPTAC database. Therefore, more prospective studies were still needed. Moreover, proteins data of this study were based on clinical specimens, which had limitations for clinical application. It would be clinically valuable, if we could discover tumor biomarkers in various accessible blood samples.

## Conclusion

In summary, our study screened 69 differentially expressed proteins in five cancers. Then, we confirmed these DEPs were mainly associated with cell proliferation and division, cellular metabolism, and extracellular matrix. According to the LASSO regression method, we have determined 24 DEPs. Notably, the DEPs-based survival-predictor model could accurately predict the OS in multiple cancers. And this is the first study to utilize proteomics to construct a pan-cancer prognosis model, and the results indicated that the pan-cancer analysis may complement single cancer analysis in the identification of prognostically differentially expressed proteins.

## Supplementary Material

Supplemental Material

## Data Availability

The datasets generated and/or analyzed during the current study are available in CPTAC (https://proteomics.cancer.gov/programs/cptac) and the Protein–Protein Interaction (PPI) database was from STRING (https://string-db.org/).

## Highlights

1. 69 differentially expressed proteins (DEPs) were identified.
2. The DEPs formed an interaction network across five cancers.
3. The 24 DEPs could accurately predict the OS in multiple cancers.
