# Supplementary material for: Development of cancer prognostic signature based on pan-cancer proteomics
Source: Bioengineered. 2020 Dec 8;11(1):1368–81. doi: 10.1080/21655979.2020.1847398 (PMC8291886; doi:10.1080/21655979.2020.1847398)
Supplement: Supplemental Material [file KBIE_A_1847398_SM6883.zip › Figure caption.docx]

Figure S1: Kaplan–Meier analysis of 10 DEPs in the training cohort. (A) RRM2; (B) PLOD2; (C) MKI67; (D) MCM5; (E) CDK1; (F) FBP1; (G) FBP2; (H) ENO3; (I) GPD1; (J) ASS1. All log rank P values of 10 DEPs were lower than 0.05. DEPs, differentially expressed proteins.

Figure S2: ROC analysis of 10 DEPs in the training cohort. (A) RRM2; (B) PLOD2; (C) MKI67; (D) MCM5; (E) CDK1; (F) FBP1; (G) FBP2; (H) ENO3; (I) GPD1; (J) ASS1. DEPs, differentially expressed proteins; AUC, Area Under the Curve.
