# Supplementary figures and images for: Development of cancer prognostic signature based on pan-cancer proteomics
Source: Bioengineered. 2020 Dec 8;11(1):1368–81. doi: 10.1080/21655979.2020.1847398 (PMC8291886; doi:10.1080/21655979.2020.1847398)

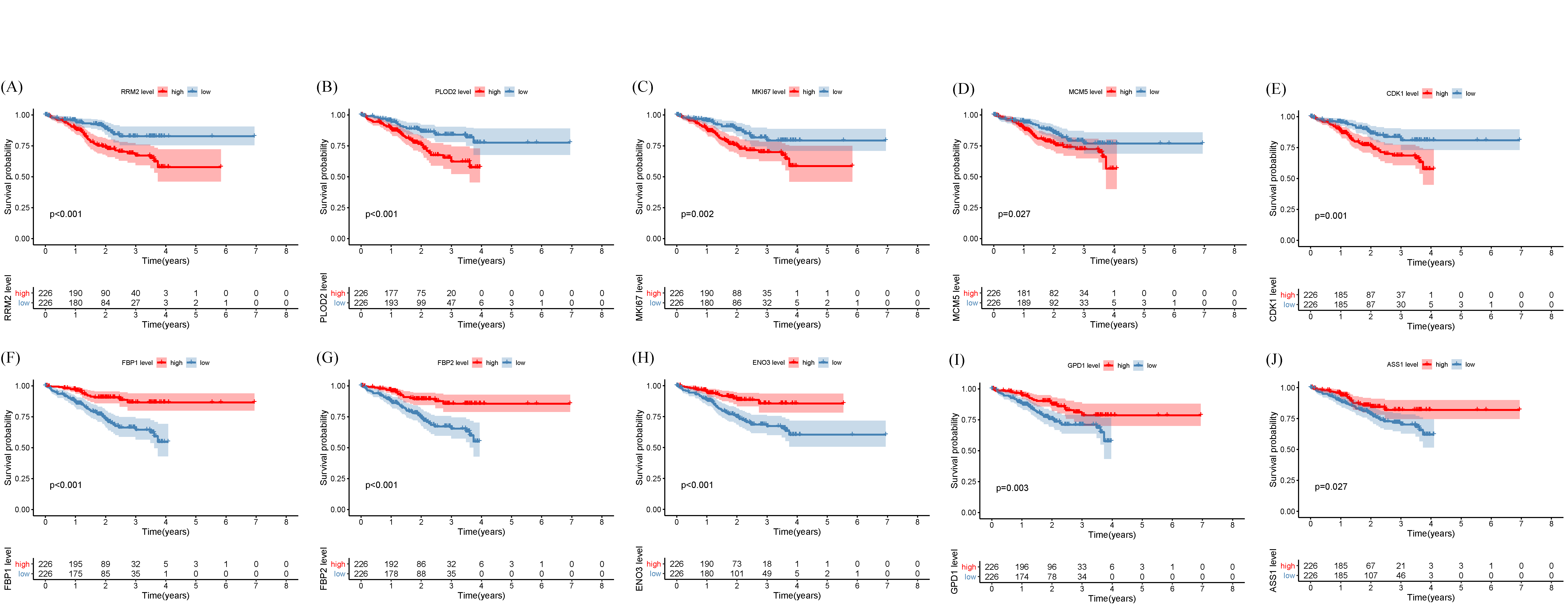

Supplement: Supplemental Material [file KBIE_A_1847398_SM6883.zip › Figure S1.tif]

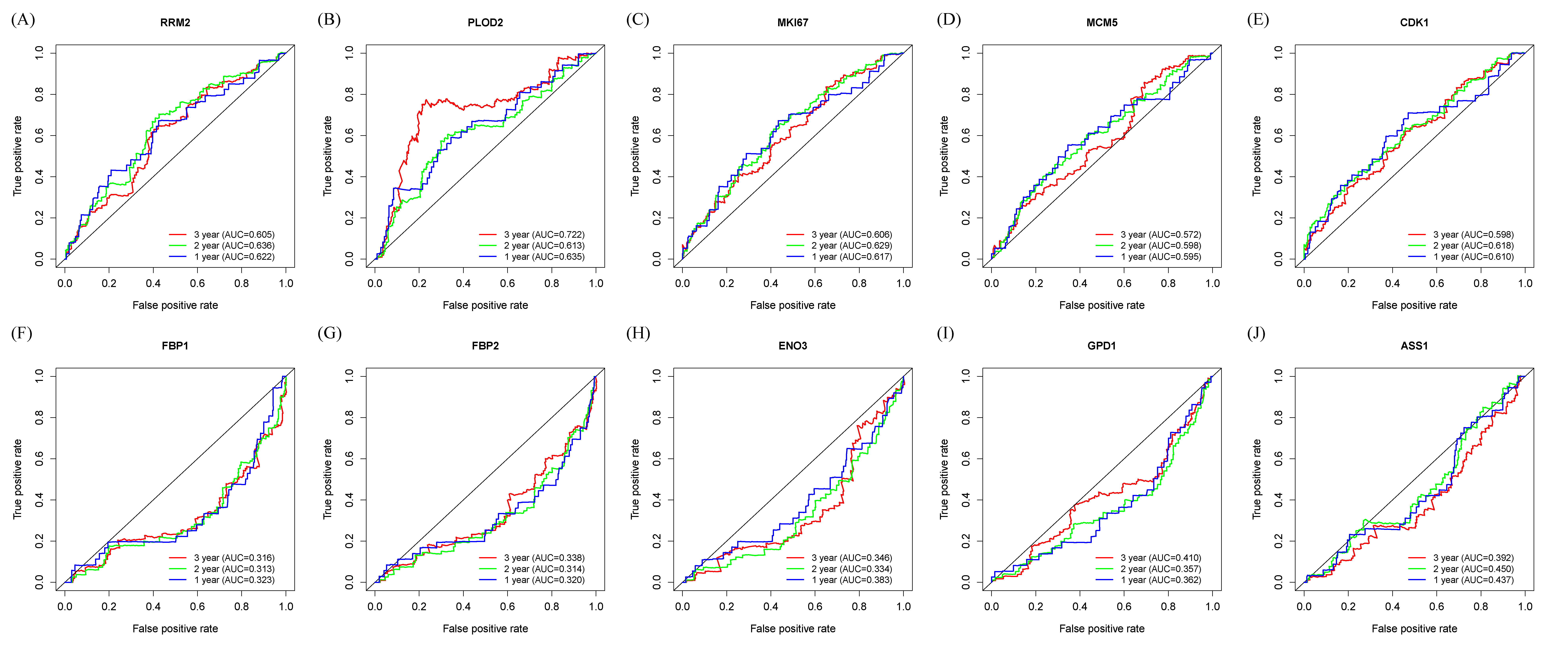

Supplement: Supplemental Material [file KBIE_A_1847398_SM6883.zip › Figure S2.tif]
